# Likelihood ratio data to report the validation of a forensic fingerprint evaluation method

**DOI:** 10.1016/j.dib.2016.11.008

**Published:** 2016-11-18

**Authors:** Daniel Ramos, Rudolf Haraksim, Didier Meuwly

**Affiliations:** a Netherlands Forensic Institute, Laan van Ypenburg 6, 2497GB The Hague, The Netherlands; b University of Twente, Drienerlolaan 5, 7522NB Enschede, The Netherlands; c ATVS – Biometric Recognition Group, Escuela Politecnica Superior, Universidad Autonoma de Madrid, C/ Francisco Tomas y Valiente 11, 28049 Madrid, Spain; d LTS5 – Signal Processing Laboratory, École Polytechnique Fédérale de Lausanne, Faculty of Electrical Engineering, Station 11, CH-1015 Lausanne, Switzerland

**Keywords:** Method validation, Automatic interpretation method, Strength of evidence, Accreditation, Validation report, Likelihood ratio data

## Abstract

Data to which the authors refer to throughout this article are likelihood ratios (LR) computed from the comparison of 5–12 minutiae fingermarks with fingerprints. These LRs data are used for the validation of a likelihood ratio (LR) method in forensic evidence evaluation. These data present a necessary asset for conducting validation experiments when validating LR methods used in forensic evidence evaluation and set up validation reports. These data can be also used as a baseline for comparing the fingermark evidence in the same minutiae configuration as presented in (D. Meuwly, D. Ramos, R. Haraksim,) [1], although the reader should keep in mind that different feature extraction algorithms and different AFIS systems used may produce different LRs values. Moreover, these data may serve as a reproducibility exercise, in order to train the generation of validation reports of forensic methods, according to [1]. Alongside the data, a justification and motivation for the use of methods is given. These methods calculate LRs from the fingerprint/mark data and are subject to a validation procedure. The choice of using real forensic fingerprint in the validation and simulated data in the development is described and justified. Validation criteria are set for the purpose of validation of the LR methods, which are used to calculate the LR values from the data and the validation report. For privacy and data protection reasons, the original fingerprint/mark images cannot be shared. But these images do not constitute the core data for the validation, contrarily to the LRs that are shared.

**Specifications Table**

**Table:** 

Subject area	*Forensic Biometrics*
More specific subject area	*Forensic Fingerprints*
Type of data	*Empirical validation report example based on real forensic fingerprint images. Likelihood ratio values computed from those real forensic fingerprints, in order to replicate the validation report.*
How data was acquired	*Fingerprints scanned using the ACCO* 1394S *live scanner, converted into the biometric scores using the Motorola BIS 9.1 algorithm.*
Data format	*Text files, Calibrated likelihood ratios supporting either H*_*p*_*or H*_*d*_*propositions*
Experimental factors	*Biometric scores were treated as per description in paragraph 4.*
Experimental features	*Same [SS] and Different [DS] source scores were produced using a Motorola AFIS comparison algorithm and used to compute the LR values as described in paragraph 5.*
Data source location	*Netherlands Forensic Institute, Laan van Ypenburg 6, 2497 GB, The Hague, The Netherlands*
Data accessibility	*Data is with the article.*

**Value of the data**

• Real forensic data in a form of LR values suitable for validation and performance evaluation are provided. The availability of LRs from forensically relevant data is limited, which increases the value of these data.
• Complete empirical validation case study presented in a form of a validation report including a validation decision is provided. The data serve for reproducibility of validation reports of automatic forensic evaluation methods as described in [1].
• The performance characteristics of the LR method developed is measured in terms of accuracy, discriminating power, calibration, generalization, coherence and robustness [1], provided in a form of calibrated likelihood ratios for both – the baseline and the multimodal LR method.

## 1 Data

The term “data” is used to denote the LR values, which are produced using two different LR methods presented below. The data are shared with the forensic biometric community, alongside with the description of an empirical example of a validation report generated using the LR values, which is included in [2]. The LR data can be used to reproduce the validation experiments for the accuracy, discriminating power and calibration in the validation report in [2]. The validation report is of potential interest of forensic researchers who aim to validate and accredit their LR systems/LR methods, and the data presented here are of use to assess the reproducibility of the results presented in the report. Presented below is an experimental design, materials, methods as well as the datasets used to produce the LR values.

## 2 Experimental design, materials and methods

In Section 3 we start off with the validation matrix in which the performance characteristics, metrics and graphical representations used are organized; introduce the similarity scores in Section 4; describe the datasets used for validation and LR method development in Section 5, define the LR methods in Section 6; define the validation criteria in Section 7; present the validation report organized in 6 tables (one per each performance characteristic) in Section 8 and conclude by introducing the validation decision in Section 9.

A more complete example of the validation report using this particular data can be found in [2].

## 3 Validation matrix

A validation report must include the specification and description of the different aspects of the validation process. Sometimes, these aspects are summarized in a so-called “Validation matrix” (Table 1).

The following aspects are essential to any validation process:

• Performance characteristic: characteristic of a LR method that is thought to have an influence in the validation of a given method. For instance, LR values should be discriminating in order to be valid, provide clear distinction between comparisons under different hypotheses. In this case, discriminating power is a performance characteristic.
• Performance metric: variable whose numeric or categorical value measures a performance characteristic. For instance, the minimum log-likelihood ratio cost (minC_llr_) can be interpreted as a measure of discriminating power, and therefore it can be used as a performance metric of the discriminating power.
• Graphical representation: representation of a performance characteristic, its distribution or its variation in the form of a graph. Note that not all graphical representations recommended in the original article [1] are included in the Validation Matrix, but at least one for each characteristic.
• Validation criteria: these define conditions for validating the method for each of the performance characteristics considered (*i.e.*, rows in the matrix). For instance, if we are measuring accuracy using Cllr as a metric, the validation criterion can be Cllr <0.2. The establishment of these criteria depends on the policy of each forensic laboratory, and should be transparent and not easily modified during the validation process. Some implications of this are discussed previously in this document.
• Data: description of the database used for validation, both in the development and in validation stages.
• Experiment: a description of the experimental protocol to generate the likelihood ratio values. Each experimental protocol might vary among different performance characteristics, especially for the secondary ones. For instance, in order to measure coherence, the protocol might significantly vary with respect to the measure of accuracy [3].
• Analytical result: value of the performance metric for the experiment. For instance, if we are measuring accuracy using Cllr as a metric, the analytical result can be Cllr=0.2. It is also often useful to express the result as a relative improvement with respect to a clearly defined baseline or reference.
• Validation decision: for each performance characteristic, the validation decision will be *pass* if the validation criterion is met by the analytical result, and *fail* otherwise.

## 4 Fingerprint evidence evaluation using AFIS scores

The method to be validated in this example is based on the output scores of an Automated Fingerprint Identification System (AFIS) comparison algorithm. The aim is to compute a likelihood ratio for each score provided by the AFIS in a comparison between a fingermark and a fingerprint. The “commercial off-the-shelf” AFIS algorithms producing comparison scores are primarily developed to support the process of selection of candidates for forensic investigation and not aimed for the process of description of the evidential value for forensic evaluation [4]. However, the information of the AFIS can be evaluated by means of a LR in order to yield complementary information to forensic examiners, especially if they are unsure about the conclusions of a comparison between a fingerprint and a fingermark. Previous work regarding this procedure can be found in [2], [5], [6], [7]. As a consequence, different methods to compute LR values from AFIS scores have been implemented and evaluated at the Netherlands Forensic Institute [2], [3], [8].

The AFIS comparison algorithm (*Motorola BIS - Printrak 9.1*) is used here as a *black box*, without the aim of scrutinizing its internal approach to compute scores. A detailed description of the algorithm inside the black box can be found in [2]. In recent work [9] it is shown that the higher the amount of scores to train the models, the more adequate the *plug-in* method.

In this example, the propositions for the computation of the LR are established at source level, and defined as follows:

• H_1_, or Same-Source (SS) proposition: The fingermark and the fingerprint originate from the same finger of the same donor.
• H_2_, or Different-Source (DS) proposition: The fingermark originates from a random finger of another donor of the relevant population, unrelated to the donor of the fingerprint.

The determination of the relevant propositions in a specific case is mandatory. However, the hypotheses determined in this particular example are generic and not intended as a recommendation in the original article [1]. They are just given for the purpose of illustration. Each particular case will lead to a different set of propositions, and this should be considered in the scope of the validation process. The determination of the hypotheses is part of the scope of the validation procedure conducted, which should be incorporated to other requirements from each particular laboratory or institution.

## 5 Datasets used

As recommended in the original article [1], different datasets are used for the development and validation stages. A “forensic” dataset, consisting of fingermarks from the real cases, was used in the validation stage. The LRs generated by the methods, are the values used to conduct the validation process, and are the data presented in this contribution.

### 5.1 Development dataset

Since it is notoriously difficult to find forensically relevant, sufficiently large datasets including the known ground truth about the origin of the specimens, we decided to use a set of simulated^1^ [9], [10] 8-minutiae^2^ fingermarks from 26 individuals paired with their corresponding fingerprints. The fingermarks were obtained by capturing an image sequence of the finger of each individual from an optical live scanner (Smiths Heimann Biometrics ACCO 1394S live scanner) and splitting the frames captured into 8 minutiae configurations.

For generating same-source (SS) scores we used the AFIS scores of simulated fingermarks and the corresponding reference fingerprint of the same finger, captured from the same individual under controlled conditions. For generating different-sources (DS) scores we used the mark in the case compared against a 200’000 - fingerprint subset of population database provided by the National Services of Dutch National Police. The number of comparisons used to generate scores is summarized in Table 2.

In order to generate an appropriate modelling of the scores for the development stage, scores are obtained on a “leave-one-person-out” basis, meaning that in the computation of a likelihood ratio from a score, the latter is eliminated from the training data for the models.

It is worth noting that, in score-based LR computation, there is some theoretical controversy about the way in which scores are computed from the training dataset (see *e.g.* [11]). However, we think that the proposed scheme to obtain scores is adequate for the sake of illustration in the original article [1], and it is by no means proposed as a recommendation for score-based systems.

### 5.2 Validation dataset

The validation dataset consists of data from real forensic cases: 58 identified fingermarks in 12-minutiae configuration and their corresponding fingerprints. The ground-truth labels of the dataset, indicating whether a fingermark/fingerprint pair originates from the same source as stated by forensic examiners is denoted as “ground-truth by proxy” because of the nature of the pairing between fingermarks and fingerprints: they have been assigned after examination by human examiners, indirectly taking into account not only the 12 minutiae, but also the correspondence of other features. The minutiae feature vectors^3^ of the fingermarks have been manually extracted by examiners while the minutiae feature vectors of the fingerprints have been automatically extracted using the feature extraction algorithm of the AFIS used, and manually checked by examiners. Those feature vectors are used to feed the AFIS comparison algorithm for the computation of scores.

In order to obtain multiple minutiae configurations for the validation of the LR method, the minutiae extracted from the fingermarks have been clustered into configurations of 5–12 minutiae, according to the method described in [10]. Following the clustering procedure, we obtain 481 minutiae clusters in a 5-minutiae configuration from the 58 fingermarks with 12 minutiae. For each cluster in the marks, a same-source (SS) score is obtained by comparing each minutiae cluster from a fingermark with the corresponding reference print. Similarly, a different-source (DS) score distribution is obtained by comparing each minutiae cluster from a fingermark to a subset of a police fingerprint database. This database consists of roughly 10 million 10-print cards captured in 500 dpi. The higher the number of minutiae in each cluster, the lower the number of clusters, as can be seen in Table 3.

### 5.3 Description of the behaviour of AFIS scores

Before the LR model under validation (and its baseline) will be introduced, an analysis of the AFIS scores is performed in order to determine the set of desirable performance characteristics (qualities) of the LR models.” Worth noting, this analysis is performed on training data, which is not used as validation database afterwards.

Additionally, the AFIS technology used employs the concept of early outs. Thus, there are three consecutive stages in each comparison:

1. Firstly, the system uses a quick comparison between the mark and the print. If the score obtained in this first comparison is −1, it is called a *first ​level early-out* and the score is delivered for that comparison, stopping the comparison process. Otherwise, a second comparison is performed.
2. If the score was not a first early-out, the AFIS does not still output the score, but performs a more sophisticated (but still fast) comparison between the mark and the print. If the score obtained is between 0 and 300 it is called a *second ​level early-out*, and it is delivered for that comparison, stopping the comparison process. Otherwise, a third level comparison is proposed.
3. If the comparison does not result in first or second early-outs, the AFIS performs a more computationally intensive comparison, where a final score bigger than 300 is finally delivered.

**Fig. 1:**
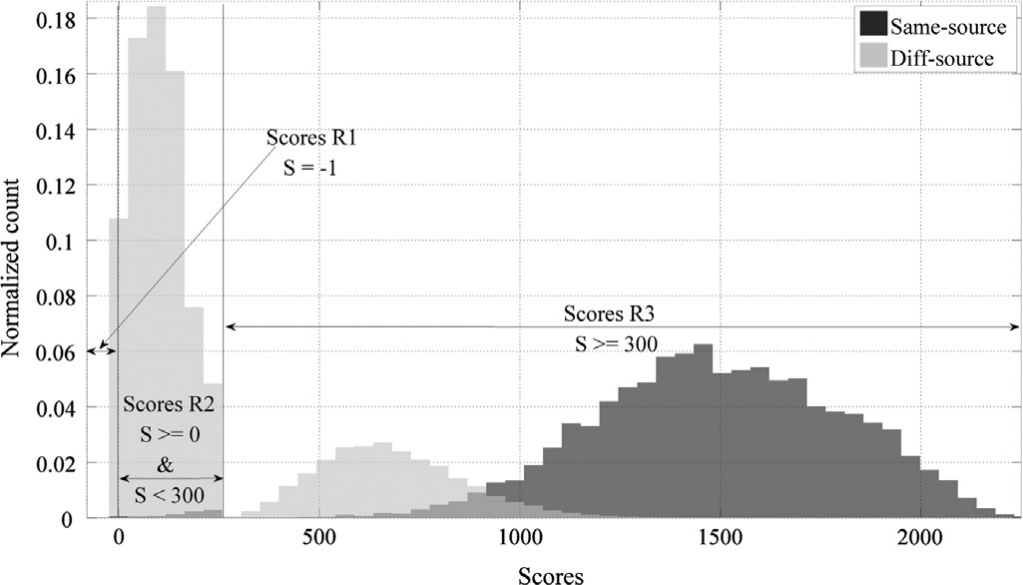
Three different regions of the scores produced by the AFIS algorithm (published in [1]).

This behaviour of the system divides the range of scores into three regions (−1, {0,300} and *more than 300*. This is shown in Fig. 1, where the scores that result from the AFIS algorithm applied to a subset of the development data are clearly distributed in those three regions (R). In Region 1 (R1) (score of −1) the first level early-outs are found. In Region 2 (R2) (scores in the {0,300} range) the second level early-outs are distributed. Finally, in Region 3 (R3) the full comparison of all the features is performed (the algorithm outputs scores bigger than 300). Additionally, it should be considered that the family of probabilistic distributions of SS and DS scores observed in each region might be different, mainly because the early-out scoring process implies the use different comparison algorithms.

The original fingerprints cannot be shared with the forensic biometric community due to restriction related to privacy and data protection. But the likelihood ratios which were produced by the two compared LR methods can be shared with the biometric community. They are the core data of the experiment, allowing to reproduce the published results.

## 6 Multimodal LR method and baseline KDF

In this section, we describe the model to validate and its baseline. The aim of the LR method to validate (the so-called multimodal method, briefly described below) is to outperform the baseline, as we discuss later. This description is needed in the validation report, if there is not a proper bibliographic reference to address it.

### 6.1 Data produced using the baseline LR method: Kernel Density Functions

The multimodal nature of the SS and DS score distributions and the non-overlap of the three regions suggests the use of flexible, non-parametric score-to-LR transformation models. A popular choice in the literature [12], [13] has been the Kernel Density Functions (KDF or KDE). For this reason, KDE will be used as the baseline model in our validation experiment. In the KDE baseline experiment we treat all the SS (and DS) scores in all three regions together to calculate LR׳s from the AFIS scores.

KDE (or any other parametric / non-parametric modelling method) will not be of much use particularly in the R1 region, since all the scores in this region have the same discrete value *S*=−1. It is an excellent example of a limitation of the use of KDE for this kind of score distribution. However, as KDE is typically chosen and recommended by many references in forensic science, and it is also theoretically grounded, we will choose it as a baseline.

Let *S* denotes the score obtained by the AFIS in the comparison between the fingermark found on the crime scene and the fingerprint of the donor. The baseline KDE LR model implements the general LR expression:

(1) $$LR=\frac{P(S|H_{p})}{P(S|H_{d})}$$

where for the fingerprint evidence evaluation datasets are defined in the following way:

• *S*_*ss*_ – a set of scores obtained from comparing a training set of simulated fingermarks of the donor with the reference fingerprint of the donor. They will be used to fit the KDE probability density in the numerator.
• *S*_*ds*_ – scores obtained from comparing the crime scene fingermark and a subset of fingerprints from the population database used in the model (in this case a subset of the operational AFIS database of the National Unit of the Dutch Police). They will be used to fit the KDE probability density in the denominator.

This approach has been proposed in [12], [13], [14], and has been dubbed *asymmetric anchoring* [8], [11]. As mentioned before, there is some discussion about the usage of the databases in score-based likelihood ratio computation [8], [11], the selection of the asymmetric anchoring as a procedure to generate the scores should not be seen as a recommendation, and discussions about this are outside the scope of this example. However, we will use it in this example as a choice for data usage in order to compute scores for training the models, just for the sake of illustration in the original article [1]. The outcomes of this method are two sets of LR values, supporting either the H_p_ or H_d_.

### 6.2 Data produced using the Multimodal LR model

In order to obtain the LR for a given score, the proposed multimodal LR model to be validated in this example independently assigns probabilities to each score region by *regional models*, and then combines them by following the rules of probability. A detailed description of the method to compute LRs can be found in [15].

As a result of the application of the LR model, one LR per comparison in the validation process is generated. Both for development and validation. The resulting set of LRs constitute the data included in this contribution.

## 7 Validation criteria

The validation criteria are established with respect to the results of the performance characteristic of the baseline method, as mentioned in Table 4 below.

## 8 Validation report

In this section, we present a validation report following the EN ISO/IEC 17025:2005 recommendations, where all the items in the validation matrix above are addressed (Table 4). The report is presented per performance characteristic in 8.1, 8.2, 8.3, 8.4, 8.5, 8.6 below.

### 8.1 Accuracy

**Fig. 2:**
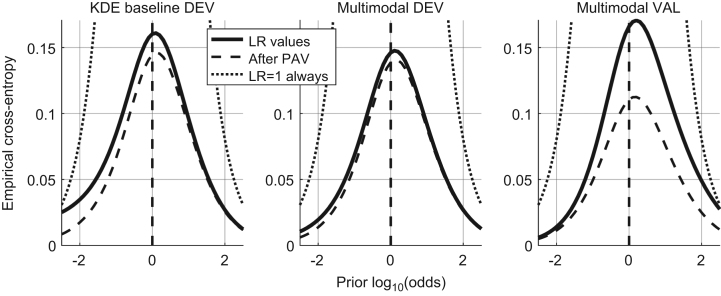
ECE plots of the KDE baseline method and the Multimodal method on the development dataset and the ECE plot of the Multimodal method on the validation dataset.

In [1] defined as “the closeness of agreement between an assigned LR and the ground truth status of the proposition in a decision-theoretical framework”. It is measured by the Cllr and represented by the ECE plot, as shown in Fig. 2.

#### 8.1.1 Validation criterion

Validation criterion for accuracy is based on the Kernel Density Function (KDE) baseline LR method. Using the development dataset in 8 minutiae configuration, Cllr=0.16 for the baseline.

Better or comparable Cllr value on the development dataset in 8 minutiae configuration is expected for the multimodal LR method than for the KDE baseline (e.g. Cllr<=0.16).

#### 8.1.2 Experiment

The Cllr (solid line in the ECE plot) is measured for both methods – KDE baseline and the multimodal LR – on the development and validation datasets.

#### 8.1.3 Data

Development dataset consists of fingermarks in 8 minutiae configuration, corresponding fingerprints, reference subset of operational police database. Validation dataset consists of the fingermarks in 8 minutiae configuration and corresponding fingerprints originating from the real forensic casework.

#### 8.1.4 Analytical results

Cllr KDE baseline method development dataset=0.16.
Cllr multimodal LR method development dataset=0.14.
Cllr multimodal LR method validation dataset=0.165.

#### 8.1.5 Validation decision for the accuracy

Based on the results presented the validation criterion was satisfied.

### 8.2 Discriminating power

**Fig. 3:**
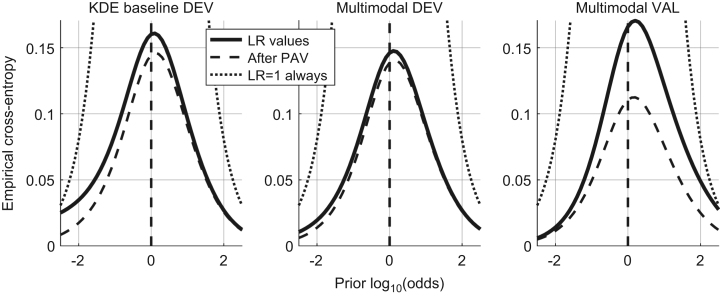
ECE plots of the KDE baseline method and the Multimodal method on the development dataset and the ECE plot of the Multimodal method on the validation dataset.

**Fig. 4:**
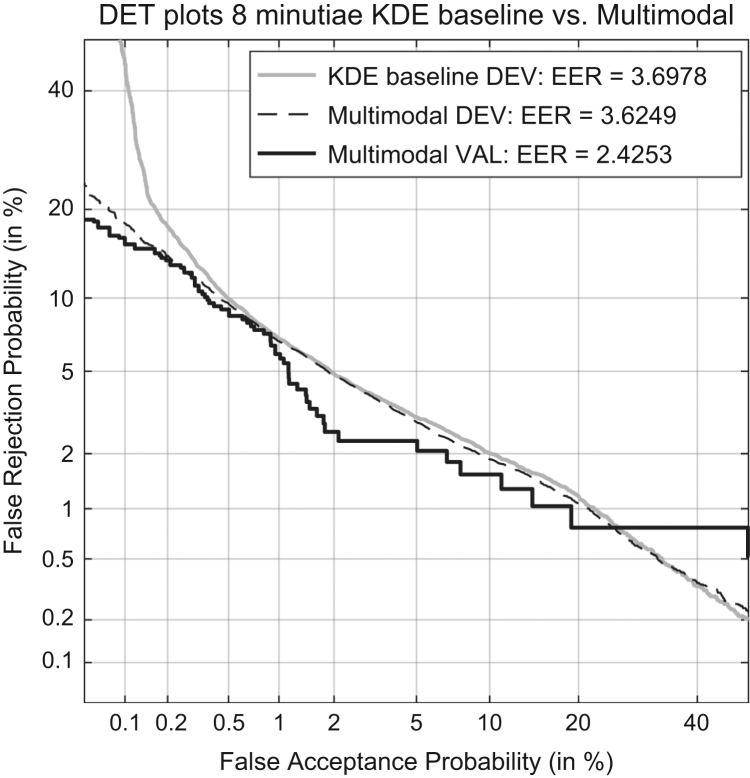
DET plots of the KDE baseline method and the Multimodal method on the development dataset and the DET plot of the Multimodal method on the validation dataset.

In [1] defined as “representing the capability of a given method to distinguish amongst forensic comparisons under each of the propositions involved”. It is measured by Cllr^min^ and EER and represented by the ECE and DET plots, as shown in Figs. 3 and 4 respectively.

#### 8.2.1 Validation criterion

Validation criterion is based on the Kernel Density Function (KDE) baseline LR method. Using the development dataset in 8 minutiae configuration, Cllr^min^=0.145 and EER=3. 7% for the baseline method.

Better or comparable multimodal LR method Cllr^min^ and EER values on the development dataset in 8 minutiae configuration are expected than the KDE baseline.

#### 8.2.2 Experiment

The Cllr^min^ (the dashed line in the ECE plot) and EER is measured for both methods – KDE baseline and the multimodal LR – on the development and validation datasets.

#### 8.2.3 Data

Development dataset consists of fingermarks in 8 minutiae configuration, corresponding fingerprints, reference subset of operational police database. Validation dataset consists of the fingermarks in 8 minutiae configuration and corresponding fingerprints originating from the real forensic casework.

#### 8.2.4 Analytical results

Cllr^min^ KDE baseline method development dataset=0.145.
Cllr^min^ multimodal LR method development dataset=0.14.
Cllr^min^ multimodal LR method validation dataset=0.11.

EER (KDE) baseline method development dataset=3.7%.
EER multimodal LR method development dataset=3.62%.
EER multimodal LR method on the validation dataset=2.4%.

#### 8.2.5 Validation decision for the discriminating power

Based on the results presented the validation criterion was satisfied.

### 8.3 Calibration

**Fig. 5:**
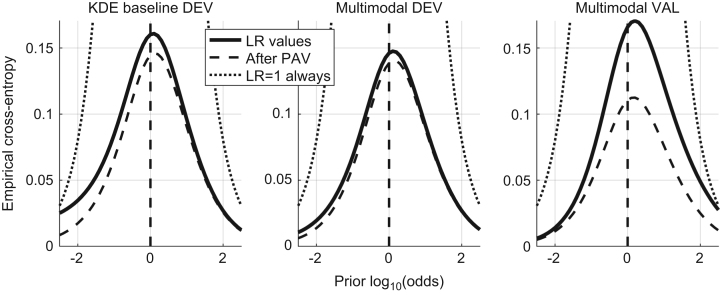
ECE plots of the KDE baseline method and the Multimodal method on the development dataset and the ECE plot of the Multimodal method on the validation dataset.

In [1] defined as “the property of a given set of LR values to yield the same set of LR values when computing the LR trained from the same data (in other words, the LR of the LR is the LR for a given set of LR values)”. It is measured by Cllr^cal^ and represented by the ECE plot, as shown in Fig. 5.

#### 8.3.1 Validation criterion

Validation criterion for accuracy is based on the Kernel Density Function (KDE) baseline LR method. Using the development dataset in 8 minutiae configuration Cllr^cal^=0.02 for the baseline method. Hence we defined the calibration criterion as Cllr^cal^ (val)≤Cllr^cal^ (dev)+0.1.

#### 8.3.2 Experiment

The Cllr^min^ is measured for both methods – KDE baseline and the multimodal LR – on the development and validation datasets.

#### 8.3.3 Data

Development dataset consists of fingermarks in 8 minutiae configuration, corresponding fingerprints, reference subset of operational police database. Validation dataset consists of the fingermarks in 8 minutiae configuration and corresponding fingerprints originating from the real forensic casework.

#### 8.3.4 Analytical results

Cllr^cal^ KDE baseline method development dataset=0.02.
Cllr^cal^ multimodal LR method development dataset=0.01.
Cllr^cal^ multimodal LR method validation dataset=0.06.

#### 8.3.5 Validation decision for the calibration

Based on the results presented the validation criterion was satisfied.

### 8.4 Robustness to the lack of data

**Fig. 6:**
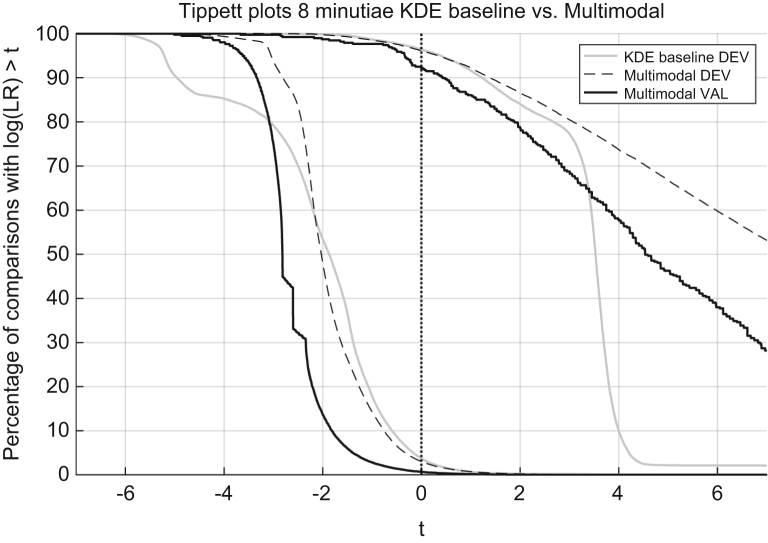
Tippett plots of the KDE baseline method and the Multimodal method on the development dataset and the ECE plot of the Multimodal method on the validation dataset.

In [1] defined in a following way. “Data driven LR methods do have a tendency to provide LR values of inappropriate magnitude when the data used to train them is not enough. Inappropriate (not suitable) LR methods may result in LR values of huge magnitudes, which given the limited amount of data cannot resemble reality.” It is observed for a range of LR values and represented in a Tippett plot, as shown in Fig. 6.

#### 8.4.1 Validation criterion

Multimodal LR method yields LR values that present moderate weight-of-evidence for the values in the baseline KDE that are extremely high (see [2] page 84).

#### 8.4.2 Experiment

The range of the LR values is analysed in search of LR values of large magnitude.

#### 8.4.3 Data

Development dataset consists of fingermarks in 8 minutiae configuration, corresponding fingerprints, reference subset of operational police database. Validation dataset consists of the fingermarks in 8 minutiae configuration and corresponding fingerprints originating from the real forensic casework.

#### 8.4.4 Analytical results

The KDE baseline methods yields evidence of enormous magnitudes supporting the wrong proposition (in extreme cases bigger than 10^90) {shown in [1] page 84}, as opposed to the method proposed, in which the support to the wrong proposition is much more confined (not bigger than 10^9 in a single extreme case). Hence the multimodal LR method developed is more robust to the lack of data than the KDE baseline method.

#### 8.4.5 Validation decision for the calibration

Based on the results presented the validation criterion was satisfied.

### 8.5 Coherence

**Fig. 7:**
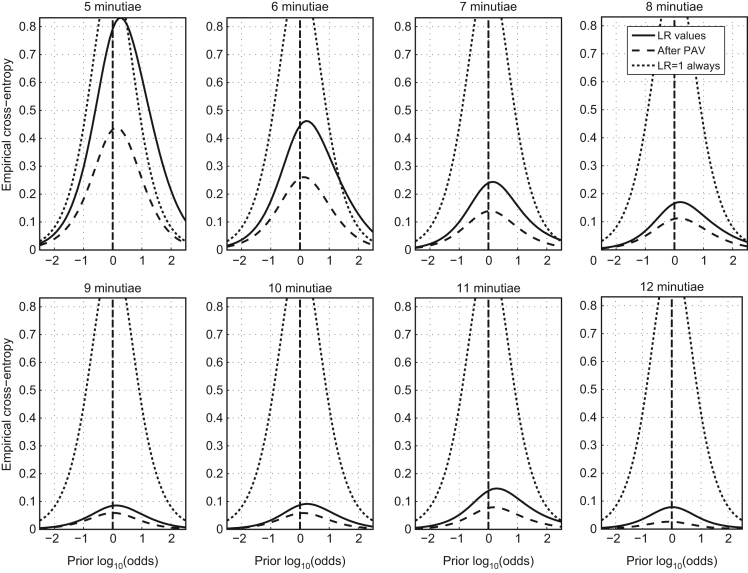
ECE plots of the Multimodal method on the validation dataset in the varying minutiae configurations (published in [3]).

**Fig. 8:**
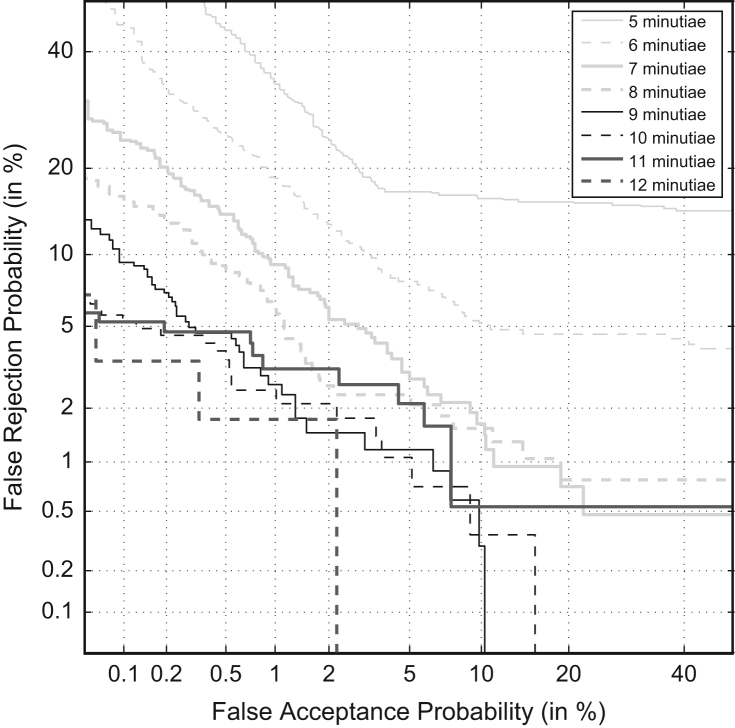
DET plots of the Multimodal method on the validation dataset in the varying minutiae configurations (published in [3]).

In [1] defined as “measures the agreement in the variation of performance metrics (Cllr, EER) when the amount of information in the evidence varies, like the quantity of minutiae in a fingerprint and a fingermark.” It is measured using the Cllr, Cllr^min^ and the EER and represented in a ECE and DET plots, as shown in Figs. 7 and 8 respectively.

#### 8.5.1 Validation criterion

Observe improvement in the performance metrics (accuracy and discriminating power) with the increasing number of minutiae (presenting additional information).

#### 8.5.2 Experiment

Vary the number of minutiae from 5 to 12 minutiae and observe improvement in Cllr, Cllr^min^ and EER.

#### 8.5.3 Data

Multimodal LR method was trained using the development dataset. Validation dataset consists of the fingermarks in 5 to 12 minutiae configurations and corresponding fingerprints originating from the real forensic casework.

#### 8.5.4 Analytical results

Table 5.

#### 8.5.5 Validation decision for the calibration

Based on the results presented the validation criterion was satisfied with the following remark:

There are two different algorithms at the AFIS minutiae comparison algorithm. The first algorithm is used for comparing fingermarks in 5 to 9 minutiae configuration; the second algorithm is used for comparing fingermarks in 10+ minutiae configuration.

This makes the coherence to fail in the transition between algorithms. However, this is a consequence of the AFIS black-box technology and not a consequence of the LR method, because the discriminating power is also affected by this, and not only the calibration.

Therefore, the proposed method clearly shows coherence within each of the algorithms. In order to show full coherence, it would be beneficiary to replace the twin-cored comparison algorithm by a dedicated minutiae comparison algorithm that would work across the whole range of minutiae configurations. However, as the use of this particular AFIS algorithm is specified in the scope of the validation process, we conclude with the accomplishment of the coherence.

### 8.6 Generalization to the previously unseen data under the dataset shift

**Fig. 9:**
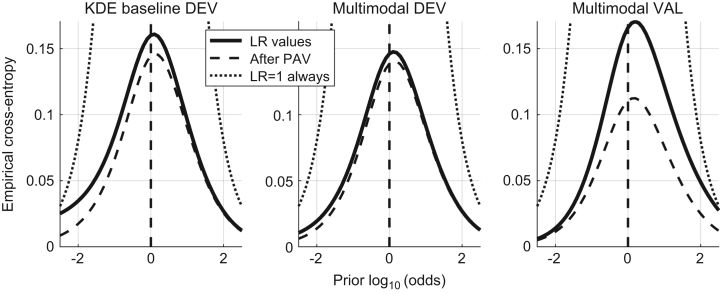
ECE plots of the KDE baseline method and the Multimodal method on the development dataset and the ECE plot of the Multimodal method on the validation dataset.

**Fig. 10:**
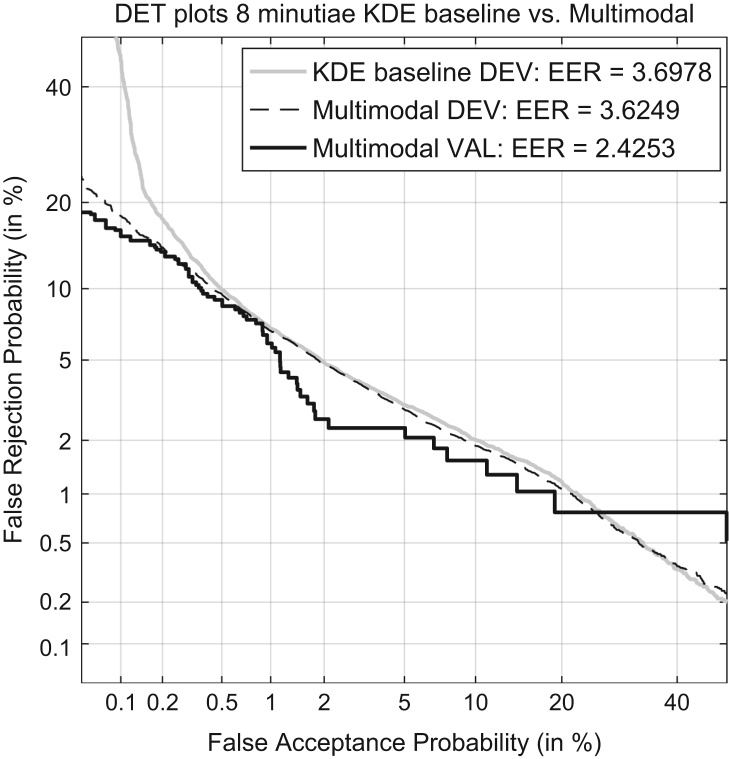
DET plots of the KDE baseline method and the Multimodal method on the development dataset and the ECE plot of the Multimodal method on the validation dataset.

In [1] defined as the “capability of a method to keep its performance under dataset shift, which is here defined as the difference in the conditions between the training data (used to train the LR methods) and the data that will be used as evidence in operational conditions.” It is measured using the Cllr, Cllr^cal^, Cllr^min^ and the EER and represented in a ECE and DET plots, as shown in Figs. 9 and 10 respectively.

#### 8.6.1 Validation criteria

Cllr (*validation* dataset)≤Cllr (*development* dataset)+0.1.
Cllr^cal^ (*validation* dataset)≤Cllr^cal^ (*development* dataset)+0.1.
Cllr^min^ (*validation* dataset)≤Cllr^min^ (*development* dataset)+0.1.
EER (*validation* dataset)≤EER (*development* dataset)+5%.

#### 8.6.2 Experiment

Multimodal LR method is trained using the development dataset and tested using the previously unseen validation dataset. An example using fingermarks in 8 minutiae configuration is used. The baseline LR method is trained using the development dataset, the Multimodal LR method trained using the development dataset and in the end the Multimodal LR method validated using the previously unseen validation dataset.

#### 8.6.3 Data

Development dataset consists of fingermarks in 8 minutiae configuration, corresponding fingerprints, reference subset of operational police database. Validation dataset consists of the fingermarks in 8 minutiae configuration and corresponding fingerprints originating from the real forensic casework.

#### 8.6.4 Analytical results

Table 6 and Table 7.

#### 8.6.5 Validation decision for the generalization to the previously unseen data

Based on the results presented the validation criteria were satisfied.

## 9 Validation decision

The multimodal LR method developed for the forensic fingerprint evidence evaluation appears to be satisfying the validation criteria specified above, with a remark regarding the coherence. Summary across different performance characteristics is presented in Table 8 below.

## Figures and Tables

**Table 1:** Aspects of empirical validation organized in a validation matrix.

Performance characteristic	Performance metric	Graphical representation	Validation criteria	Data	Experiment	Analytical result	Validation decision
Accuracy	Cllr	ECE plot	According to the definition	Data used	Description	+/− [%] compared to the baseline	Pass/fail
							
Discriminating power	EER, Cllr^min^	ECE^min^ plot	According to the definition	Data used	Description	+/− [%] compared to the baseline	Pass/fail
		DET plot					
							
Calibration	Cllr^cal^	ECE plot	According to the definition	Data used	Description	+/− [%] compared to the baseline	Pass/fail
		Tippett plot					
							
Robustness	Cllr, EER,	ECE plot	According to the definition	Data used	Description	+/− [%] compared to the baseline	Pass/fail
	
	Range of the LR	DET plot					
		Tippett plot					
							
Coherence	Cllr, EER	ECE plot	According to the definition	Data used	Description	+/− [%] compared to the baseline	Pass/fail
		DET plot					
		Tippett plot					
							
Generalization	Cllr, EER	ECE plot	According to the definition	Data used	Description	+/− [%] compared to the baseline	Pass/fail
		DET plot					
		Tippett plot					

**Table 2:** Same and different source scores.

**Individual**	Comparisons for SS scores	Comparisons for DS scores
**Person 1**	8’455 marks – 1 print	8’455 marks – 200’000 prints
**Person 2**	2’751 marks – 1 print	2’751 marks – 200’000 prints
**Person 3**	4’666 marks – 1 print	4’666 marks – 200’000 prints
**Person 4**	2’206 marks – 1 print	2’206 marks – 200’000 prints
**Person 5**	3’179 marks – 1 print	3’179 marks – 200’000 prints
**Person 6**	3’758 marks – 1 print	3’758 marks – 200’000 prints

**Table 3:** Validation dataset sizes for SS and DS scores. Note that the number of SS scores is the same as the number of clusters for a given minutiae number.

	SS scores	DS scores
5 minutiae	481	10’283’780
6 minutiae	432	9’236’160
7 minutiae	426	9’107’880
8 minutiae	387	8’274’060
9 minutiae	342	7’311’960
10 minutiae	286	6’114’680
11 minutiae	190	4’062’200
12 minutiae	58	1’240’040

**Table 4:** Validation criteria. First 3 columns of the Validation Matrix used in this example. Note that not all metrics recommended in [1] are included in the Validation Matrix, but at least one of it for each characteristic.

Performance characteristic	Performance metric	Validation criteria (from KDE Baseline)
Accuracy	Cllr	Cllr better (lower of equal) than the baseline
Discriminating power	Cllr^min^ EER	Cllr^min^ and EER better (lower of equal) than the baseline
Calibration	Cllr^cal^	Cllr^cal^ (val)≤Cllr^cal^ (dev) + 0.1
		
Robustness to the lack of data	Cllr, EER	Tippett plots present better behaviour of extreme LR values than the baseline
	Range of LR values	
		
Coherence	Cllr, EER	Cllr_12min_< Cllr_11min_
		…
		Cllr_6min_< Cllr_5min_
		EER_12min_< EER_11min_
		…
		EER_6min_< EER_5min_
		
Generalization	Cllr, EER	Cllr^min^ (val) <=Cllr^min^ (dev)+0.1
		Cllr^cal^ (val) <=Cllr^cal^ (dev)+0.1
		Cllr (val) <=Cllr (dev)+0.1
		EER (val) <=EER (dev)+5%

**Table 5:** Results for the Accuracy and discriminating power with varying number of minutiae in the fingermarks of the validation dataset.

**#Minutiae**	Discriminating power		Accuracy
	**EER**	**Cllr**^**min**^	**Cllr**
5 minutiae	15.9	0.43	0.5
6 minutiae	6.9	0.26	0.28
7 minutiae	3.9	0.14	0.16
8 minutiae	2.4	0.11	0.13
9 minutiae	1.5	0.063	0.075
10 minutiae	2.2	0.063	0.074
11 minutiae	2.7	0.081	0.1
12 minutiae	1.8	0.057	0.084

**Table 6:** KDE baseline vs. multimodal LR method trained on the development dataset.

Dataset	Cllr^min^	Cllr^cal^	Cllr	EER
KDE baseline development	0.145	0.02	0.16	3.7
Multimodal validation	0.11	0.06	0.165	2.43

**Table 7:** Multimodal LR method trained on the development dataset and validated on the validation dataset.

Dataset	Cllr^min^	Cllr^cal^	Cllr	EER
Multimodal development	0.14	0.01	0.146	3.62
Multimodal validation	0.11	0.06	0.165	2.43

**Table 8:** Validation decisions across different performance characteristics.

Performance characteristic	Validation decision
Accuracy	Pass
Discrimination	Pass
Calibration	Pass
Robustness	Pass
Coherence	Pass
	*with remark
Generalization	Pass

## Footnotes

1 Simulated fingermarks in this case refer to series of image captions of a finger moving on a glass plate of the fingerprint scanner (the procedure is described in detail in [10]).

2 Please note that the performance characteristics of the LR model described in Section 6 have been evaluated using the development dataset based on the fingermarks in the 8 minutiae configuration (which is the quality threshold for usability of fingermark evidence in some countries). Subsequently the LR model was validated using the validation dataset for a range of 5–12 minutiae configuration fingermarks.

3 Minutiae feature vectors of a fingermark or fingerprint in our case consist of feature type, position, and orientation (parallel to the ridge flow).
